# Development of a Mucoadhesive In Situ Gelling Formulation for the Delivery of *Lactobacillus gasseri* into Vaginal Cavity

**DOI:** 10.3390/pharmaceutics11100511

**Published:** 2019-10-03

**Authors:** Barbara Vigani, Angela Faccendini, Silvia Rossi, Giuseppina Sandri, Maria Cristina Bonferoni, Pietro Grisoli, Franca Ferrari

**Affiliations:** Department of Drug Sciences, University of Pavia, V.le Taramelli, 12, 27100 Pavia, Italy; barbara.vigani@unipv.it (B.V.); angela.faccendini01@universitadipavia.it (A.F.); giuseppina.sandri@unipv.it (G.S.); cbonferoni@unipv.it (M.C.B.); pietro.grisoli@unipv.it (P.G.); franca.ferrari@unipv.it (F.F.)

**Keywords:** vaginal candidosis, *L. gasseri*, in situ thermogelling vehicle, mucoadhesion, poloxamer, methylcellulose, pectin, xyloglucan

## Abstract

Local administration of vaginal probiotics, especially lactobacilli, has been recently proposed as an effective prevention strategy against candidosis recurrences, which affect 40–50% of women. In this context, the aim of the present work was the development of a mucoadhesive in situ gelling formulation for the vaginal administration of *Lactobacillus gasseri*. Mixtures of poloxamer 407 (P407) and methylcellulose (MC), two thermosensitive polymers, were prepared and subjected to rheological analyses for the assessment of their sol/gel transition temperature. The association of P407 (15% *w*/*w*) with MC (1.5% *w*/*w*) produced an increase in gelation extent at 37 °C even after dilution in simulated vaginal fluid (SVF). The presence of 0.5% *w*/*w* pectin (PEC) produced a reduction of vehicle pH and viscosity at 25 °C that is the vehicle resistance to flow during administration. The presence of a low concentration of xyloglucan (XYL) (0.25% *w*/*w*) increases the mucoadhesive properties and the capability to gelify at 37 °C of the formulation after dilution with SVF. A three-component (P407/MC/PEC; 3cM) and a four-component (P407/MC/PEC/XYL; 4cM) mixture were selected as promising candidates for the delivery of *L. gasseri* to the vaginal cavity. They were able to preserve *L. gasseri* viability and were cytocompatible towards the HeLa cell line.

## 1. Introduction

Vulvovaginal candidosis (VVC) is an opportunistic infection of vaginal mucosa, mainly caused by *Candida sp.* that represents one of the most frequent causes of gynecologic counseling. Candidosis symptoms, even though not related to high mortality, negatively affect the patient quality of life: VVC is frequently associated to vaginal inflammation (vaginitis) with irritation and pruritus, dysuria and dyspareunia, a “cottage cheese-like” vaginal discharge, vulvar burning and fissuring, often accompanied by mucosal lesions and slight bleeding [1,2]. Lactobacilli are an important component of vaginal microflora since they produce lactic acid, responsible for the low vaginal physiological pH. A change in vaginal microbiota composition in terms of a reduction of lactobacilli species favors the overgrowth of pathogens like Candida [3]. 

Candidosis affects 70–75% of women at least once in their life, particularly during reproductive age; furthermore, it has been reported that 40–50% of women experience one or more recurrences after the apparent resolution of the first infective episode. Most recurrences may be caused by the persistency of some yeast strains in the vaginal lumen despite antifungal treatment, along with some predisposing factors, rather than exogenous reinfections [4,5]. 

Since highly effective antimycotic treatments are available for the management of acute infections, but not for preventing recurrences, the use of vaginal probiotics, such as lactobacilli, have been explored in the contest of VVC [6,7]. Lactobacilli exert a prophylactic effect by maintaining or restoring the physiological equilibrium of the complex vaginal ecosystem through a wide variety of mechanisms of action. Vaginal probiotics are able to directly compete with pathogens for both nutrients and adhesion sites at the mucosal surface, to produce hydrogen peroxide, bio-surfactants and antimicrobial metabolites, in addition to organic acids (lactic and formic acids), which contribute to lower intra-vaginal pH, thus establishing a hostile environment for the growth of pathogens [3,7,8,9].

Among *Lactobacillus* sp., *L. gasseri* represents one of the predominant colonizer of the vaginal milieu in the highest number of women. Maldonado-Barràgan et al. [10] demonstrated that some strains of *L. gasseri* could be used as effective probiotics in preventing vaginal bacterial infections by producing some antimicrobial compounds, named bacteriocins, able to directly inhibit pathogen colonization into the vaginal lumen. More recently, other authors proved that the supernatants collected from *L. gasseri* and *L. crispatus* cultures inhibited the initial colonization and maturation of *C. albicans* biofilm. Moreover, such supernatants produced the down-regulation of the expression of all genes, related to biofilm formation, and the inhibition of *C. albicans* adhesion to HeLa cells [11].

A crucial issue in the local administration of therapeutic agents for the treatment of vaginitis is the formulation of suitable delivery systems, able to reside at the site of infection for a prolonged period of time in order to achieve the therapeutic effects. In the last decades, mucoadhesion and thermogelling approaches have been proposed to improve in vivo performance of vaginal formulations [12,13,14,15,16,17,18,19,20,21]. At room temperature (20 °C), the low viscosity of the formulation allows an easy administration in the vaginal cavity and an optimal spreading on the mucosa. An increase in temperature (20 °C→37 °C) promotes the in situ gelation of the administered formulation, which becomes resistant to the removal mechanisms of the vaginal environment, ensuring a long permanence of the loaded therapeutic agent at the site of infection [22]. Among the polymers used for the preparation of thermogelling systems, poloxamers, synthetic co-polymers of poly(ethylene oxide-propylene oxide-ethylene oxide), have been widely investigated due to their thermo-reversible behavior in aqueous solution [20,23]. They are declared as GRAS excipients for several pharmaceutical applications, due to their excellent compatibility, and they are able to guarantee a prolonged release of the active ingredients [24]. On the other hand, poloxamer-based thermogelling systems are characterized by poor mucoadhesion properties. To overcome this disadvantage, hydrophilic polymers have been added to poloxamer formulations in order to improve their interaction with the mucosa, prolonging the retention time in the vaginal cavity [20,23,25]. 

Given these premises, the aim of the present work was the development of an extemporaneous in situ gelling formulation for the vaginal administration of *L. gasseri* for preventing candidosis recurrences. The vehicle should exhibit the following features: (i) acidic pH to create an hostile vaginal environment to the persistence of some *Candida* sp.; (ii) ability to gelify at 37 °C to guarantee a prolonged (12–24 h) presence of probiotics at the site of infection; (iii) low viscosity at room temperature to promote an easy administration and an optimal spreadability on the vaginal mucosa; (iv) mucoadhesive behavior; (v) biocompatibility with the loaded lactobacilli; (vi) cytocompatibility. 

To this aim, mixtures of thermosensitive and mucoadhesive polymers were considered: poloxamer (P407) and methylcellulose (MC) were selected as thermogelling agents and xyloglucan (XYL) was used as bioadhesive and moisturizing agent [26,27]. Pectin (PEC) was added to the formulation as acidifying agent. The occurrence of a synergistic effect among polymers in terms of rheological properties and mixture capability to interact with vaginal mucosa was investigated. Afterwards, two formulation prototypes were selected and their ability to preserve the viability of the loaded lactobacilli was studied. 

## 2. Materials and Methods 

### 2.1. Materials

Poloxamer P407 (P407) (Kolliphor P407, BASF SE, Ludwigshafen, Germany), low methoxy pectin (PEC) (Giusto Faravelli, Milan, Italy), xyloglucan/HS (XYL) (Indena SpA, Milan, Italy) and methylcellulose (MC) (Methocel A4M, Colorcon, UK) were used for the preparation of the in situ gelling formulations.

Simulated vaginal fluid (SVF) was prepared using bovine serum albumin (BSA), calcium hydroxide, glucose and urea, purchased from Sigma-Aldrich (Milan, Italy), potassium hydroxide, acetic acid 96% *v*/*v*, glycerol, hydrochloric acid 37% *v*/*v* and sodium chloride, from Carlo Erba (Milan, Italy) and lactic acid 80% *v*/*v* from A.C.E.F. (Fiorenzuola d’Adda, Piacenza, Italy). 

Porcine gastric mucin type II (Sigma-Aldrich, Milan, Italy) was used as biologic substrate for the evaluation of formulation mucoahedesive properties. 

For experiments with HeLa cells, the materials hereafter reported were used. Dimethyl sulfoxide (DMSO), Dulbecco’s Phosphate Buffer Solution (PBS), MTT (3-(4,5-dimethylthiazol-2-yl)-2,5-diphenyltetrazolium bromide), antibiotic/antimycotic solution (100×; stabilized with 10,000 units penicillin, 10 mg streptomycin, and 25 μg amphotericin B per mL), trypan blue solution and trypsin-EDTA solution were purchased from Sigma-Aldrich (Milan, Italy). Dulbecco’s Modified Eagles Medium (DMEM) with 4.5 g/L glucose and l-Glutamine (DMEM-HG) was purchased from Corning Incorporated (Corning, NY, USA) and inactivated fetal bovine serum (FBS) from Biowest (Nuaillé, France).

### 2.2. Preparation of Thermosensitive Formulations 

A P407 solution was prepared in distilled water and maintained under magnetic stirring at 4 °C until a clear solution was obtained. PEC was separately solubilized in distilled water at room temperature and, then, added to P407 solution at 4 °C in order to obtain two polymeric mixtures with the following compositions: 15% *w*/*w* P407/0.2% *w*/*w* PEC and 15% *w*/*w* P407/0.5% *w*/*w* PEC. 

An MC solution was prepared by dispersing MC in distilled water under magnetic stirring at 80–85 °C to allow the complete polymer hydration corresponding to the attainment of a clear polymer solution. Subsequently, the MC solution was blended, at room temperature, with P407/PEC mixture in order to obtain a three-component mixture (3cM) having the following composition: 15% *w*/*w* P407/0.5% *w*/*w* PEC/1.5% *w*/*w* MC. 

An XYL solution was prepared by dissolving XYL in distilled water under magnetic stirring at 70–75 °C and MC solution, prepared as mentioned above, was added at the same temperature to XYL. A four-component mixture (4cM) was prepared, at room temperature, blending XYL/MC mixture with P407/PEC one; it is composed of 15% *w*/*w* P407/0.5% *w*/*w* PEC/0.75% *w*/*w* MC/0.25% *w*/*w* XYL. 

The pH of each formulation was measured by means of a microprocessor pH meter (Hanna Instruments, Padua, I).

### 2.3. Preparation of Thermosensitive Formulations Diluted in Simulated Vaginal Fluid 

Simulated vaginal fluid (SVF) was prepared according to Chang et al. [12]. Briefly, urea 0.04% *w*/*v*, sodium chloride 0.351% *w*/*v*, potassium hydroxide 0.14% *w*/*v*, lactic acid 0.25% *w*/*v*, acetic acid 0.104% *w*/*v*, glucose 0.5% *w*/*v*, Ca(OH)_2_ 0.022% *w*/*v*, glycerol 0.016% *w*/*v* and bovine serum albumin 0.002% *w*/*v* were dissolved in distilled water. The pH of the solution was adjusted to 4.2 with HCl 4N.

All formulations were diluted with SVF. In particular, 10:1 and 10:1.5 formulation: SVF *w*/*w* ratios were used in order to mimic the dilution to which formulations could undergo after vaginal administration [28].

### 2.4. Rheological Measurements

The rheological analyses were carried out by means of a rotational rheometer (MCR 102, Anton Paar, Turin, Italy) equipped with a cone plate combination (CP50-1, diameter = 50 mm; angle = 1°) as measuring system. Before each analysis, thermosetting time was fixed at 300 s.

#### 2.4.1. Viscosity Measurements

All samples, undiluted and after dilution in SVF, were subjected to viscosity measurements. Sample viscosity was measured in a temperature range of 25–40 °C, by applying a shear rate of 5 s^−1^. Such shear rate was chosen because at low shear rates all the samples are characterized by higher viscosity values (showing a pseudoplastic behavior), which the instrument is able to measure with a high accuracy. 

3cM and 4cM were also subjected to viscosity analyses at room temperature (25 °C) at increasing shear rates (10–300 s^−1^). Three replicates were effected for each sample.

#### 2.4.2. Viscoelastic Measurements

Sample viscoelasticity was assessed by dynamic oscillatory measurements, such as stress sweep test and oscillation test. In the stress sweep test, increasing stresses were applied at a constant frequency (0.1 Hz) and the elastic response of the sample, expressed as storage modulus G′, was measured. Such a test allows the identification of the “linear viscoelastic region”. In the oscillation test, a shear stress value, chosen in the linear viscoelastic region previously determined, was applied and G′ (storage modulus) and G″ (loss modulus) profiles were recorded. 

In particular, 15% *w*/*w* P407 solution and P407/PEC mixtures were subjected to oscillation measurements at 1 Hz in the temperature range 25–40 °C to identify the sample gelation temperature. Moreover, 3cM and 4cM were subjected to oscillation measurements at 25 °C and increasing frequency values ranging from 0.1 to 10 Hz. The same test was carried out at 37 °C after sample dilution with SVF. 

Three replicates were considered for each sample.

### 2.5. Mucoadhesion Measurements 

The mucoadhesive properties of P407/MC, 3cM and 4cM mixtures, after dilution in SVF at 10:1.5 *w*/*w* ratio, were assessed at 37 °C by means of a TA-XT plus Texture Analyzer (Stable Micro Systems, Godalming, UK) equipped with an 1 kg load cell and a cylindrical movable probe (P/10C). A dispersion of porcine gastric mucin (8% *w*/*w*) was prepared in SVF and used as biological substrate [23].

Thirty µL of each sample (mixture diluted according to 10:1.5 *w*/*w* ratio) was layered on a filter paper disc (Ø = 10 mm) and fixed on the movable probe. Thirty µL of mucin dispersion were fixed, faced to the formulation, on the sample holder. After 300 s, the probe was lowered to put in contact mucin dispersion with the diluted vehicle. A preload of 2500 mN was applied for 300 s. Then, the probe was raised at a constant speed (2.5 mm/s) up to the complete mucin-sample detachment. Blank measurements were carried out using 30 µL of SVF instead of mucin dispersion. Six replicates were effected for each sample.

The maximum detachment force (Fmax) was measured and the differential parameter ΔFmax was calculated according to the following equation:

ΔFmax = Fmax_mucin_ − Fmax_blank_ (1)

where Fmax_mucin_ was the maximum force measured in presence of mucin and Fmax_blank_ the maximum force measured in absence of mucin (blank measurements). 

The mucoadhesive properties of P407/MC, 3cM and 4cM mixtures, after dilution in SVF at 10: 1.5 *w*/*w* ratio, were also evaluated by means of an *ex vivo* detachment test, using porcine vaginal mucosa (2 cm × 2 cm flap) as biological substrate. Each sample (30 μL) was layered on a filter paper disc (Ø = 30 mm), glued to the mobile probe, which was put in contact with porcine vaginal mucosa obtained by a slaughterhouse (animals weighed 130–140 kg and were 1–2 years old). Porcine vaginal mucosa was deprived of the connective tissue and fixed, using a cyanoacrylate glue, to the sample holder facing the sample. In order to allow the formation of mucoadhesive joints, a preload of 2500 mN was applied for 300 s. The movable probe was then raised at a constant speed (2.5 mm/min) up to the complete mucosa-formulation detachment. The Fmax was measured. Six replicates were carried out for each sample.

### 2.6. Probiotic Viability Assay 

Microbiological analyses were carried out to evaluate the viability of *L. gasseri* after loading into both 3cM and 4cM vehicles. *L. gasseri*, purchased from American Type Culture Collection (ATCC: 33323), was cultured in De Man, Rogosa and Sharpe Agar (MRS Broth, Oxoid, Basingtoke, UK) at 37 °C for 48 h in anaerobic conditions. After incubation, lactobacilli suspension was centrifuged (3500 rpm) for 20 min. The supernatant was removed and lactobacilli pellet was washed with saline solution. The microbial concentration was checked to obtain a microbial suspension of ~10^6^ CFU/mL. 

The lactobacilli suspension (10^6^ CFU/mL) was added in a 1:5 *v*/*v* ratio to both 3cM and 4cM mixtures, previously prepared in aseptic conditions, and incubated for 0, 3, 6 and 24 h at 37 °C 5% CO_2_ atmosphere. A microbial suspension prepared in PBS in the same above-mentioned *v*/*v* ratio was considered as control. At each time point, the microorganism viability upon contact with vehicles was evaluated and expressed as CFU/mL. Three replicates were effected for each lactobacilli loaded-mixture and for the control.

### 2.7. Formulation Cytocompatibility Towards HeLa Cells

The cytotoxic effect of 3cM and 4cM mixtures, previously prepared under aseptic conditions, was investigated on HeLa cells. HeLa cells (ATCC^®^ CCL-2^™^, American Type Culture Collection, Manassas, Virginia, USA) were cultured in polystyrene flasks (Cellstar^®^ tissue culture flasks, Greiner bio-one, PBI International, Milan, Italy) in complete culture medium, consisting of DMEM-HG supplemented with 10% *v*/*v* FBS (Biowest, Nuaillé, France) and 1% *v*/*v* antibiotic-antimycotic solution. Cells were incubated at 37 °C in 5% CO_2_ atmosphere. After reaching sub-confluence (85% of flask plastic bottom covered), HeLa cells were detached every 3 days from the flasks by trypsinization and, subsequently, were seeded in polystyrene flasks or in a Transwell^®^ 12-well plate (Cellstar^®^ permeable support tissue culture plate 12 wells, 12 mm insert, sterile, polystyrene plate Greiner Bio-One, Milan, Italy), which is a permeable support characterized by a microporous membrane. Such a system was used because it enables to avoid the direct contact between the vehicle and the HeLa monolayer: the incubation temperature (37 °C), in fact, promotes the gelation of the vehicle that, if placed in direct contact with cells, could induce cell sufferance, thus affecting the results of the cytotoxicity assay. The Transwell^®^ insert divided each well in a basolateral chamber and an apical one. 

Briefly, 1.5 × 10^5^ cells/well were seeded in the basolateral chamber of each well in presence of complete culture medium (CM) and left in incubator in order to reach confluence (100% of flask plastic bottom covered). After 24 h, the medium was removed and HeLa cell monolayer was rinsed with PBS. Subsequently, 1.5 mL of fresh CM were added in the basolateral chamber of each well, and 500 μL of (a) 3cM mixture diluted 1:4 *v*/*v* or 1:9 *v*/*v* in SVF, (b) 4cM mixture diluted 1:4 *v*/*v* or 1:9 *v*/*v* in SVF and (c) CM were placed in the apical chamber. CM was used as positive control. After incubation (24 h, 37 °C, 5% CO_2_), Transwell^®^ supports, containing the vehicles, were removed and a MTT assay was performed in order to evaluate the viability of HeLa cells. CM was removed from the basolateral chamber, cell monolayers were washed with PBS and, then, 250 μL of MTT 7.5 μM in 500 μL of DMEM without phenol red were added to each well and incubated for 3 h. Finally, 2.4 mL of DMSO, used as solubilizing agent, was added to each well in order to promote the complete dissolution of formazan crystals, obtained from MTT dye reduction by mitochondrial dehydrogenases of alive cells. The solution absorbance was measured by means of an iMark^®^ Microplate reader (Bio-Rad Laboratories S.r.l.) at a wavelength of 570 nm and 690 nm (reference wavelength) after 60 s of mild shaking. Results were expressed as % viability by normalizing the absorbance measured after contact with vehicle/SVF to that measured for CM (control).

Eight replicates were effected for each sample.

### 2.8. Statistical Analysis

Whenever possible, experimental results obtained by the various types of measurements were subjected to statistical analysis, carried out by means of the statistical package Statgraphics 5.0 (Statistical Graphics Corporation, Rockville, MD, USA). In particular, a one-way ANOVA-multiple range test was used. 

## 3. Results and Discussion

### 3.1. Rheological Properties

**Figure 1:**
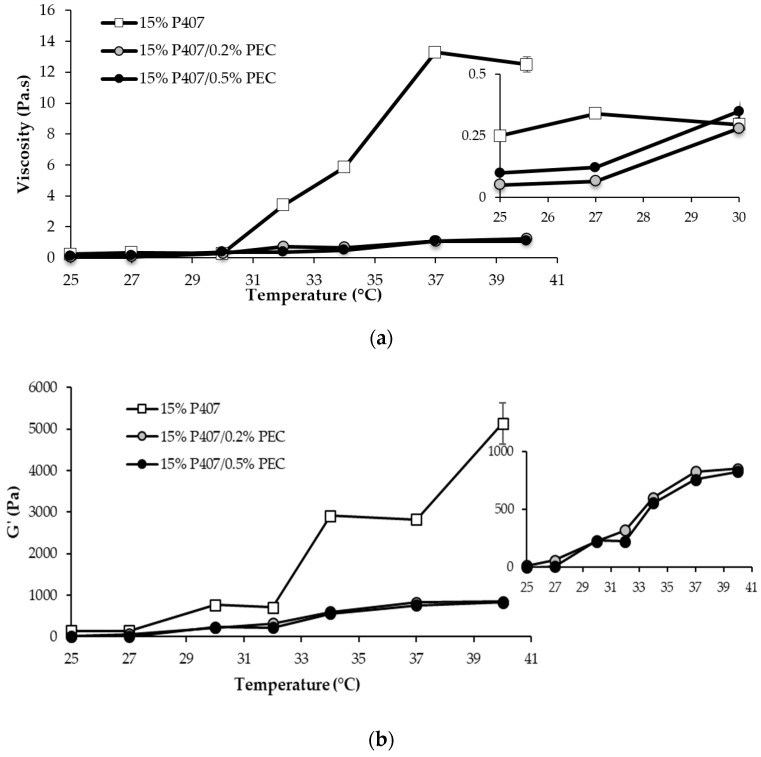
Viscosity (at 5 s^−1^ shear rate) (**a**) and G′ (at 1 Hz) (**b**) vs. temperature profiles of 15% *w*/*w* P407 solution and its mixtures with PEC (0.2 and 0.5% *w*/*w*) (mean values ± s.d.; *n* = 3).

In Figure 1, viscosity (a) and G′ modulus (b) vs. temperature profiles of 15% *w*/*w* P407 solution and its mixtures with PEC are reported.

A sharp increase in P407 solution viscosity is observed for temperature values higher than 30 °C, indicating P407 capability to gelify as a result of temperature variation (Figure 1a). The use of thermosensitive polymers, characterized by a gelation temperature close to the physiological one, represents an effective approach for the preparation of drug delivery systems intended for vaginal administration. In particular, a low viscosity at room temperature should ensure an easy administration and a homogeneous spreading of the formulation on the mucosa [29,30,31]. The gelation occurring in the vaginal cavity should render the formulation more resistant to the physiological removal mechanisms and to dilution in vaginal secretions, thus guarantying its prolonged permanence at the administration site [20,21]. 

It is well known that poloxamers are amphiphilic polymers and show a thermo-reversible behavior in aqueous solution at concentrations above a critical value, known as critical micellar concentration (CMC). In Figure 1a, the viscosity vs. temperature profile of 15% *w*/*w* P407 solution suggests that, at temperatures higher than 30 °C, the aggregation of poloxamer molecules into spherical micelles, constituted by a core of dehydrated hydrophobic propylene oxide (PO) and by a shell of hydrated hydrophilic ethylene oxide (EO), results in system gelation. 

The addition of PEC, independently of the concentration, produces a lowering of the gelation temperature and a marked reduction of the increase in sample viscosity on temperature increase (Figure 1a). The rheological behavior of P407/PEC mixtures demonstrates that the presence of co-solutes in poloxamer aqueous solutions could affect micellization and, thus, the sol/gel transition phenomenon. This result is in line with what reported in the literature [32]. In particular, the addition of PEC, which is a hydrocolloid capable to entrap water and to form gels at concentrations even lower than 1% *w*/*w* [33], may determine a rearrangement of water molecules bound to PO or EO residues and, thus, a decrease of the water content of the micellar core. The micellization is therefore favored, with a reduction of the critical micellar temperature (CMT) and, in turn, a lowering of the gelation temperature. 

PEC, an anionic polysaccharide, acts as acidifying agent when mixed with P407. In fact, the pH values of P407 mixtures, containing PEC at 0.2 and 0.5% *w*/*w* concentrations, are equal to 3.73 and 3.27, respectively. In case of vaginal candidosis recurrences, the administration of formulations characterized by an acidic pH is functional to create a hostile environment for the persistence of some *Candida* sp. [2].

The thermogelling behavior of P407-based solutions is also proved by oscillatory measurements (Figure 1b). A steep increase in G′ vs. temperature profiles is generally recognized as an index of sample gelation [20]. Since the results obtained from oscillatory test (Figure 1a) were in line with that obtained from viscosity measurements (Figure 1b), in the continuation of the work, sample structural changes caused by a temperature increase were investigated by means of viscosity measurements.

**Figure 2:**
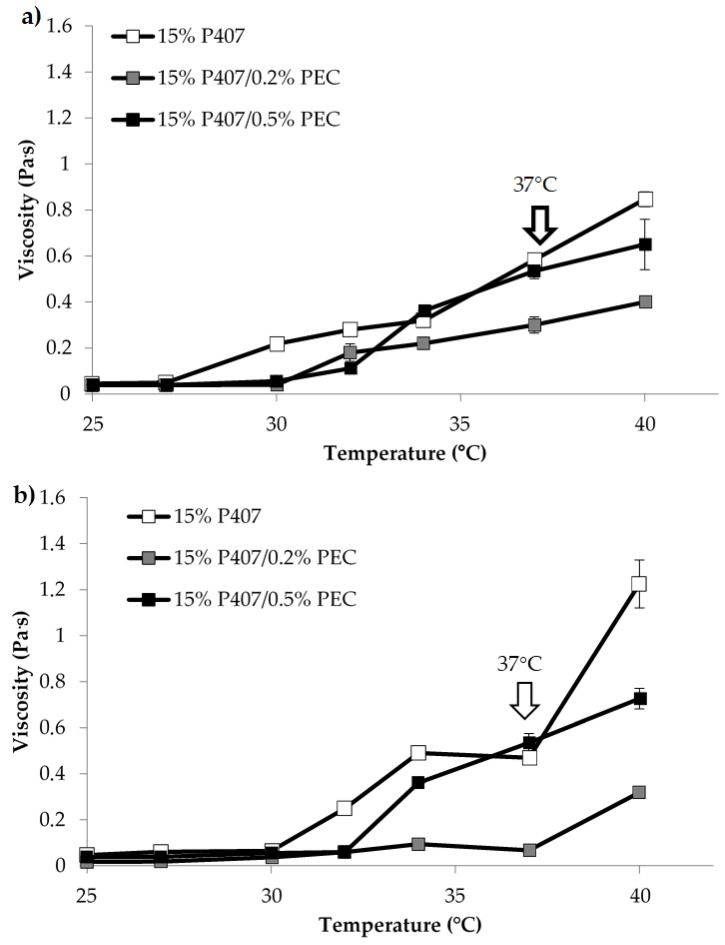
Viscosity (at 5 s^−1^ shear rate) vs. temperature profiles of 15% *w*/*w* P407 solution and its mixtures with PEC (0.2 and 0.5% *w*/*w*) upon dilution in SVF: (**a**) 10: 1 *w*/*w*; (**b**) 10:1.5 *w*/*w* (mean values ± s.d.; *n* = 3).

Since the formulation undergoes dilution in the vaginal fluids upon administration, it seemed interesting to evaluate the viscosity of the samples upon dilution in SVF according to two different weight ratios, 10:1 and 10:1.5 *w*/*w* (Figure 2). Such ratios were calculated considering a formulation volume of 5 mL and a vaginal fluid volume of 0.5–0.75 mL, as reported in the literature [20]. 

In Figure 2, viscosity vs. temperature profiles of 15% *w*/*w* P407 solution and its mixtures with PEC upon dilution in SVF according to 10:1 and 10:1.5 *w*/*w* ratios are reported. The comparison between the results reported in Figure 2 and Figure 1a reveals that the dilution in SVF leads to a lowering of the viscosity vs. temperature profile of P407 solution, which is characterized by viscosity values of the same order of magnitude of those observed for P407/PEC mixtures subjected to the same dilution. Such a result could be explained by the fact that P407 gelation mechanism occurs only when poloxamer concentration is above a critical value (CMC) [20]. 

Moreover, 15% *w*/*w* P407/0.5% *w*/*w* PEC mixture shows at 37 °C, independently of dilution, higher viscosity value than that observed for 15% *w*/*w* P407/0.2% *w*/*w* PEC mixture (Figure 2). The rheological analyses demonstrated that PEC at the concentration of 0.5% *w*/*w*, when blended with P407, has the capability to modulate the gelation temperature of the formulation after dilution in SVF. On the basis of these results, 0.5% *w*/*w* was selected as the optimal PEC concentration for the preparation of further formulations.

Since MC is able to gelify in aqueous solution on increasing temperature [34,35,36,37], it seemed interesting to verify if the presence of MC could improve the rheological response of the samples. 

**Figure 3:**
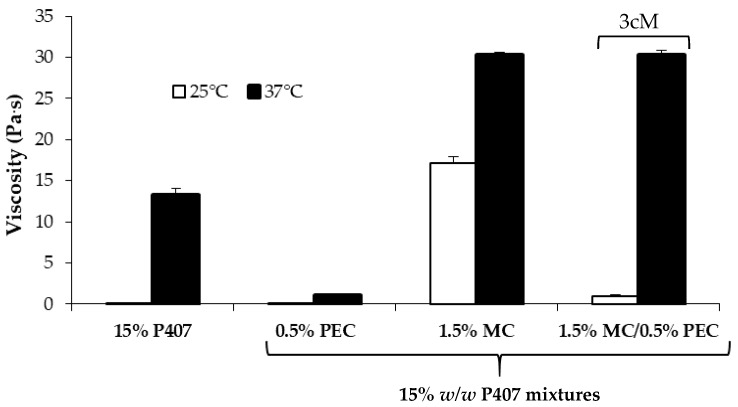
Viscosity values (at 5 s^−1^ shear rate) of 15% *w*/*w* P407 solution and its mixtures with PEC (0.5 *w*/*w*), MC (1.5% *w*/*w*) and 1.5% *w*/*w* MC/0.5% *w*/*w* PEC (mean values ± s.d.; *n* = 3).

In Figure 3 viscosity values (at 5 s^−1^ shear rate) of 15% *w*/*w* P407 solution and its mixtures with PEC (0.5 *w*/*w*), MC (1.5% *w*/*w*) and 1.5% *w*/*w* MC/0.5% *w*/*w* PEC are compared.

It can be observed that the presence of MC produces an increase in P407 viscosity at both 25 and 37 °C. In particular, the viscosity measured at 25 °C for P407/MC mixture is ten-fold higher than that of P407 solution. Since an increase in viscosity indicates an increase of sample resistance to flow, the addition of MC should not favor the administration of the formulation. On the contrary, the addition of 0.5% *w*/*w* PEC to P407/MC mixture is responsible for a viscosity decrease at room temperature, without affecting the P407/MC gelation properties at 37 °C, which remains significantly higher than that of P407 alone. The three-component mixture containing 15% *w*/*w* P407, 1.5% *w*/*w* MC and 0.5% *w*/*w* PEC is indicated with the acronym 3cM.

**Figure 4:**
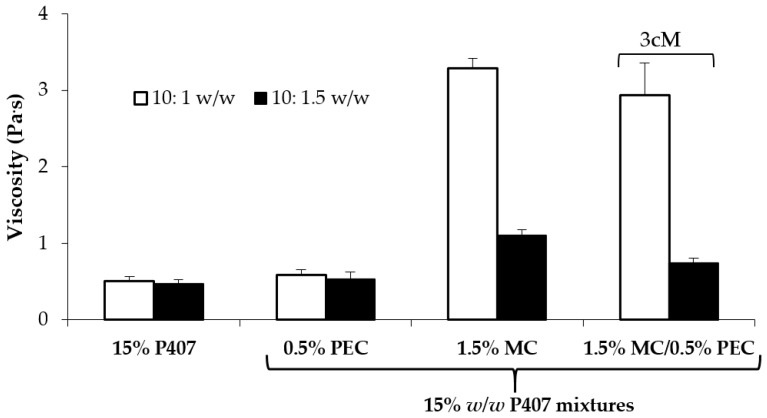
Viscosity values (at 5 s^−1^ shear rate and 37 °C) of 15% *w*/*w* P407 solution and its mixtures with PEC (0.5 *w*/*w*), MC (1.5% *w*/*w*) and 1.5% *w*/*w* MC/0.5% *w*/*w* PEC (3cM), upon dilution in SVF according to 10: 1 and 10:1.5 *w*/*w* ratios (mean values ± s.d.; *n* = 3).

In Figure 4, viscosity values of all the tested samples measured at 37 °C upon dilution in SVF are compared. It can be observed that, even after dilution, the 3cM mixture is characterized at 37 °C by viscosity values significantly higher than those of P407 solution and P407/PEC mixture and comparable to those of P407/MC mixture and by a low viscosity at 25 °C (Figure 3). The same mixture is also characterized by a pH value equal to 3.65, suitable for vaginal administration.

It is reported in literature that MC undergoes gelation at temperature values higher than 50 °C. Recently, some authors have observed a decrease of MC gelation temperature when the cellulose derivative was mixed with hydrophilic polymers [35]. It seemed therefore interesting to investigate if the presence of XYL, a natural polymer well known for its capability to interact and hydrate mucous substrates [26], could modify the rheological properties of MC solution. 

**Figure 5:**
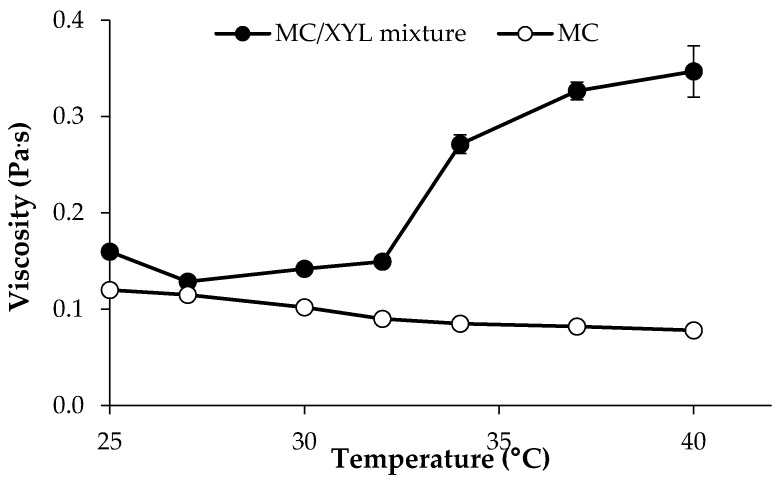
Viscosity (at 5 s^−1^ shear rate) vs. temperature profiles of 0.75% *w*/*w* MC/0.25% *w*/*w* XYL mixture and of 0.75% *w*/*w* MC solution (mean values ± s.d.; *n* = 3).

In Figure 5, viscosity vs. temperature profiles of 0.75% *w*/*w* MC/0.25% *w*/*w* XYL mixture and of the 0.75% MC solution are reported. It can be observed that XYL is able to lower MC gelation temperature, which stands around 32 °C.

On the basis of these results, it was decided to prepare a mixture containing all the four polymers (4cM, four component mixture), P407, MC, PEC and XYL. When compared to the previously investigated three-components mixture (3cM), P407 and PEC concentrations remained unchanged, whereas a lower concentration of MC (0.75% *w*/*w*) was used with the aim of maintaining a low sample viscosity at 25 °C after the addition of 0.25% *w*/*w* XYL. The 4cM mixtures has a pH equal to 3.5.

The two mixtures, 3cM and 4cM, undiluted and diluted with SVF (10:1 and 10:1.5 *w*/*w* ratios) were subjected to a thorough rheological characterization. In particular, viscosity and oscillation measurements were performed at 25 °C on undiluted samples and at 37 °C on diluted ones. 

**Figure 6:**
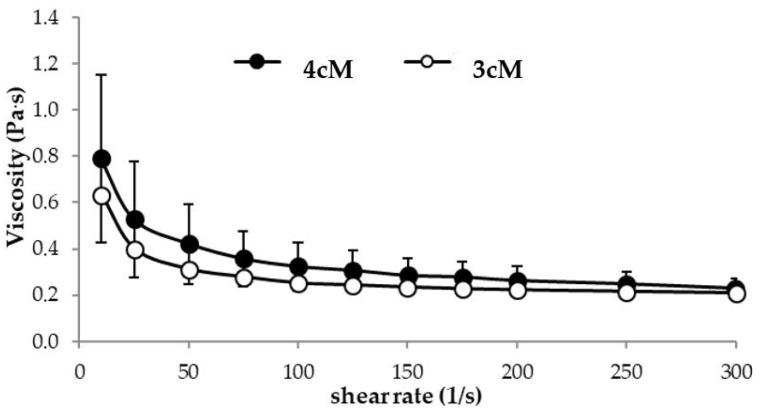
Flow curves of 3cM (15% *w*/*w* P407/1.5% MC/0.5% *w*/*w* PEC) and 4cM (15% *w*/*w* P407/0.75% *w*/*w* MC/0.5% *w*/*w* PEC/0.25% *w*/*w* XYL) mixtures, measured at 25 °C (mean values ± s.d.; *n* = 3).

In Figure 6, flow curves measured at 25 °C for 3cM and 4cM are compared. The two mixtures show comparable viscosity profiles at room temperature. In particular, they are characterized by a pseudoplastic behavior (i.e., viscosity decreases on increasing shear rate), which is functional to an easy administration: the greater the stress applied to the mixture, the lower the resistance to flow [38,39,40]. The two formulations, when subjected to a preliminary physical stability test (storage at 4 °C for 3 weeks) did not undergo any modification in terms of viscosity profiles (see Supplementary Data). 

Moreover, both 3cM and 4cM show a thermo-reversible behavior: the viscosity measured at 25 °C does not change when the samples were maintained at 37 °C for 15 min and then left to cool for 30 min at 25 °C. They are also characterized by a quick gelation time: already after 30 s at 37 °C they form a gel.

**Figure 7:**
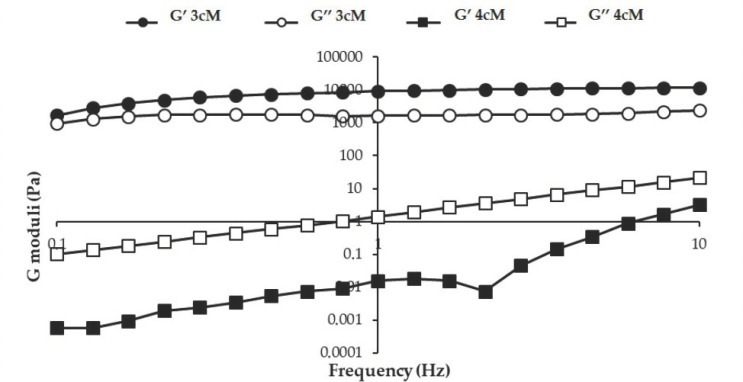
G′ and G″ moduli vs. frequency profiles of 3cM (15% *w*/*w* P407/1.5% MC/0.5% *w*/*w* PEC) and 4cM (15% *w*/*w* P407/0.75% *w*/*w* MC/0.5% *w*/*w* PEC/0.25% *w*/*w* XYL) mixtures, measured at 25 °C (mean values ± s.d.; *n* = 3).

In Figure 7, storage (G′) and loss (G″) moduli vs. frequency profiles obtained at 25 °C for the two mixtures are reported. Between the two mixtures, 3cM shows, at room temperature, greater elastic properties than 4cM. Whereas for 4cM mixture the viscous component overcomes the elastic one, 3cM mixture is characterized by a prevalence of the elastic behavior over the viscous one. For an easy administration, it is preferable that the viscous contribution is prevalent on the elastic one, i.e., the energy imparted to the sample during administration is mainly used to flow and not to recover the undergone deformation [38].

**Figure 8:**
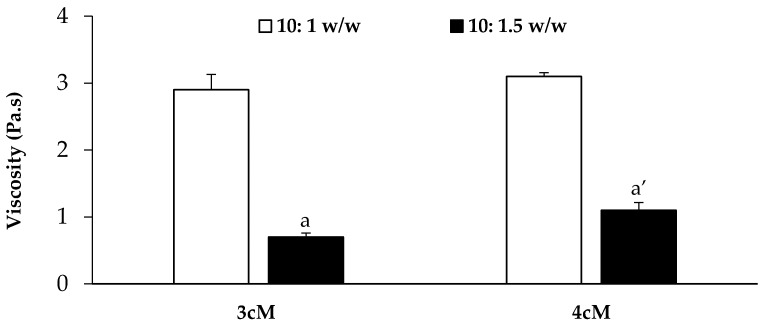
Viscosity values (at 5 s^−1^ shear rate and 37 °C) of the 3cM (15% *w*/*w* P407/1.5% MC/0.5% *w*/*w* PEC) and 4cM (15% *w*/*w* P407/0.75% *w*/*w* MC/0.5% *w*/*w* PEC/0.25% *w*/*w* XYL) mixtures upon dilution in SVF according to 10: 1 and 10: 1.5 *w*/*w* ratios (mean values ± s.d.; *n* = 3). One-way ANOVA-multiple range test (*p* < 0.05): a vs. a′.

In Figure 8, viscosity values (at 5 s^−1^ shear rate) measured at 37 °C for both mixtures upon dilution in SVF according to 10:1 *w*/*w* and 10:1.5 *w*/*w* ratios are reported. It can be observed that the presence of XYL is responsible for an increase in formulation viscosity at 37 °C, even if statistically significant only for the mixtures diluted according to 10:1.5 *w*/*w* ratio. These results are in line with the results reported by Itoh and colleagues [41], who pointed out the occurrence of a synergistic interaction between XYL and PEC, resulting in the formation of a three-dimensional polymeric network that improves the gelation properties of the polymer mixture.

**Figure 9:**
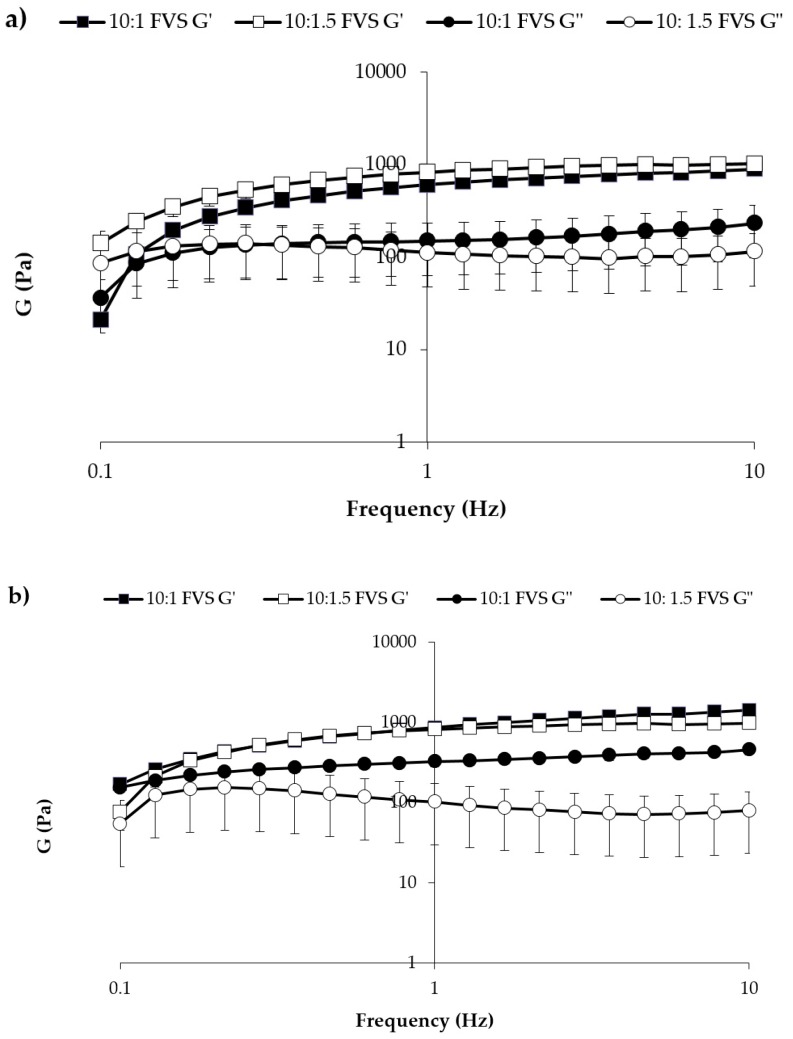
G′ and G″ vs. frequency profiles of (**a**) 3cM (15% *w*/*w* P407/1.5% MC/0.5% *w*/*w* PEC) and (**b**) 4cM (15% *w*/*w* P407/0.75% *w*/*w* MC/0.5% *w*/*w* PEC/0.25% *w*/*w* XYL) mixtures, measured at 37 °C upon dilution with SVF according to 10: 1 and 10: 1.5 *w*/*w* ratios (mean values ± s.d.; *n* = 3).

In Figure 9 the elastic storage (G′) and viscous loss (G″) moduli vs. frequency profiles are reported for both mixtures diluted in SVF according to 10:1 and 10:1.5 *w*/*w* ratios.

After dilution, both mixtures are characterized at 37 °C by G′ higher than G″ values, indicating a prevalence of the elastic on the viscous contribution. This behavior indicates the capability of the mixtures to respond elastically to a deformation (i.e., to partially recover deformation upon stress removal) and then of protecting vaginal lesions [34,35,36].

### 3.2. Mucoadhesion Properties

**Figure 10:**
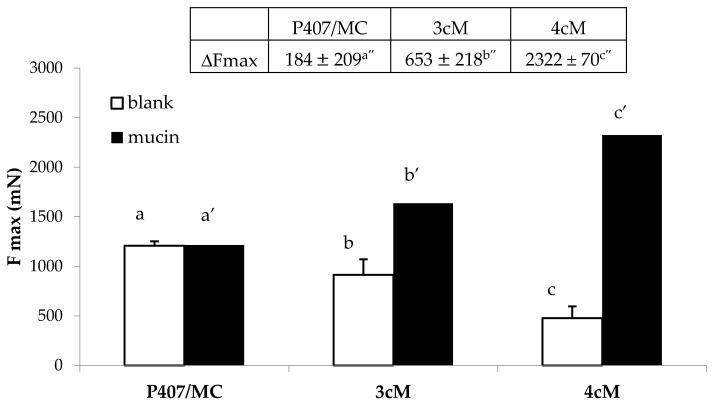
Fmax and ΔFmax values of P407/MC, 3cM (15% *w*/*w* P407/1.5% MC/0.5% *w*/*w* PEC) and 4cM (15% *w*/*w* P407/0.75% *w*/*w* MC/0.5% *w*/*w* PEC/0.25% *w*/*w* XYL) mixtures in presence and in absence (blank) of mucin (mean values ± s.d.; *n* = 6). One-way ANOVA- multiple range test (*p* < 0.05): b vs. b′; c vs. c′; a′ vs. b′, c′; b′ vs. c′; a″ vs. b″, c″; b″ vs. c″.

In Figure 10, the results of the mucoadhesion measurements performed on P407/MC, 3cM and 4cM mixtures, diluted with SVF according to 10:1.5 *w*/*w* ratio, in presence and in absence of mucin suspension are reported. It can be observed that P407/MC mixture does not show any mucoadhesive potential, being characterized by comparable Fmax values in presence and in absence (blank) of mucin (a vs. a′ not significant different). On the contrary, both 3cM and 4cM mixtures show Fmax values significantly higher in presence of mucin than those determined in absence of the biological substrate (b vs. b′ and c vs. c′ significant different), indicating their capability to interact with mucin glycoprotein chains. In presence of the biological substrate, according to mucoadhesion theory, the interaction between polymer and mucin creates an interface where the macromolecular chains of the two species are interpenetrated forming weak bonds. In this perspective, if the force value recorded in presence of the biological substrate is higher than that obtained by blank measurements (where the only contribution of the polymer is measured), the sample is characterized by a mucoadhesive potential.

The mixture containing XYL (4cM) is characterized by the highest value of the differential parameter ΔFmax (2322 ± 70 mN), followed by 3cM (653 ± 21 mN), and finally the P407/MC mixture (184 ± 20 mN). It means that 4cM mixture is characterized by the greatest mucoadhesive potential [38]. This results is clearly attributable to the presence of XYL; this polysaccharide, in fact, is referred in literature as a good mucoadhesive agent due to its mucin-like configuration with a backbone chain having branching xylose and galactoxylose substituents [42,43].

**Figure 11:**
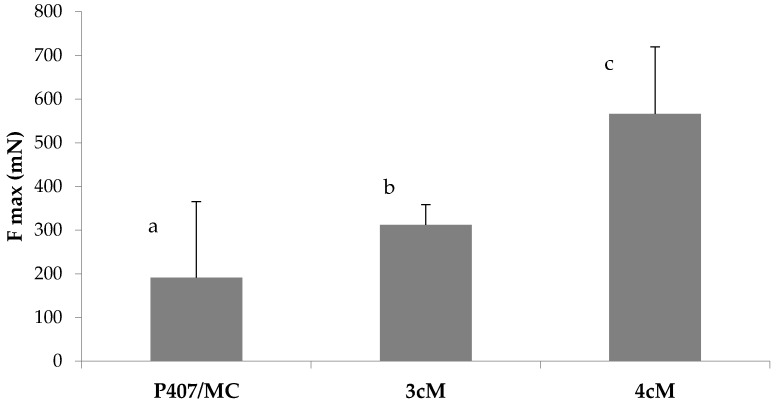
Fmax values of P407/MC, 3cM (15% *w*/*w* P407/1.5% MC/0.5% *w*/*w* PEC) and 4cM (15% *w*/*w* P407/0.75% *w*/*w* MC/0.5% *w*/*w* PEC/0.25% *w*/*w* XYL) mixtures in presence of porcine vaginal mucosa. One-way ANOVA multiple range test (*p* < 0.05): a vs. c; b vs. c.

Figure 11 reports the results of the mucoadhesion measurements carried out in presence of porcine vaginal mucosa. These results are in line with those obtained using mucin suspension as biological substrate: the best mucoadhesive properties, in fact, are shown by 4cM mixture, that is characterized by an Fmax value significantly higher than those measured for P407/MC and 3cM samples.

### 3.3. Formulation Compatibility towards L. gasseri

The final product consists of a polymeric vehicle where lactobacilli have to be extemporarily dispersed. In order to exert a preventive effect against vaginal candidosis recurrences, a high number of viable cells of *L. gasseri* have to reach and colonize the vaginal mucosa. Therefore, the microorganism viability, upon dispersion into both 3cM and 4cM mixtures and 1:5 *v*/*v* dilution in SVF, was investigated. 

In Table 1, CFU/mL of *L. gasseri* upon contact for different times with the two diluted vehicles mixtures are reported. The results obtained demonstrate that both mixtures do not affect microorganism viability up to 24 h contact.

### 3.4. Fornulation Cytocompatipility towards HeLa Cells

**Figure 12:**
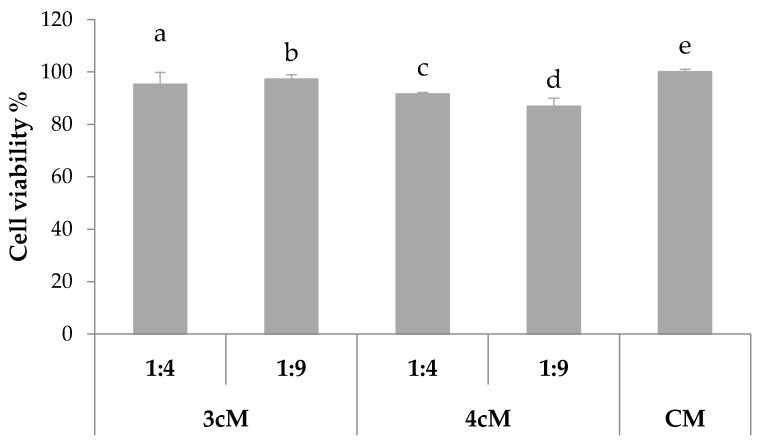
% viability of Hela cells upon contact with 3cM (15% *w*/*w* P407/1.5% MC/0.5% *w*/*w* PEC) and 4cM (15% *w*/*w* P407/0.75% *w*/*w* MC/0.5% *w*/*w* PEC/0.25% *w*/*w* XYL) mixtures (mean values ± s.d.; *n* = 8).

In Figure 12, the results of the preliminary *in vitro* cytocompatibility test performed on Hela cells are reported. It can be observed that both 3cM and 4cM mixtures, when diluted 1:4 and 1:9 *v*/*v* in SVF, show cell viability % values comparable to that of the complete culture medium, considered as control. 

These results demonstrate that the two formulations do not release in SVF any substances that could affect cell viability and, then, they can be considered biocompatible.

## 4. Conclusions

The developed formulations are promising candidates for the delivery of *L. gasseri* to vaginal cavity. They are biocompatible and able to gelify at 37 °C upon dilution in SVF and to preserve microorganism viability, while restoring a physiological vaginal environment. The results obtained point out the occurrence of a synergic action of the four polymers employed. The addition of MC to P407 solution is responsible for an increase in mixture viscosity at 37 °C upon dilution in SVF. The addition of PEC to P407/MC mixture leads to a lowering of mixture viscosity at 25 °C and guarantees acidic pH to the formulation. This behavior is functional to an ease administration and a prolonged permanence of the in situ gelling formulation on the mucosa. Moreover, the presence of XYL in 4cM formulation produces, together with the modulation of MC gelation temperature, an improvement of mucoadhesive properties. Finally, both 3cM and 4cM result to be stable in terms of rheological properties and gelation behavior up to 3 weeks at 4 °C. 

To date, a long-term physical stability test on both formulations is ongoing. *In vivo* animal tests are also planned to evaluate the safety of the formulations in terms of mucosal irritation potential.

## Figures and Tables

**Table 1:** CFU/mL of *L. gasseri* upon contact with the two mixtures and relevant controls for increasing times (CV % < 12%; *n* = 3).

SAMPLES	CFU/mL			
	0 h	3 h	6 h	24 h
**3cM**	14.73 × 10^6^	34.42 × 10^6^	46.94 × 10^6^	7950 × 10^6^
**Control (for 3cM)**	13.91 × 10^6^	32.96 × 10^6^	47.74 × 10^6^	7560 × 10^6^
**4cM**	14.69 × 10^6^	36.64 × 10^6^	48.64 × 10^6^	7716 × 10^6^
**Control (for 4cM)**	13.70 × 10^6^	36.72 × 10^6^	47.26 × 10^6^	7544 × 10^6^

## Supplementary Materials

The following are available online at https://www.mdpi.com/1999-4923/11/10/511/s1, Figure S1: Viscosity curves of 3cM (15% *w*/*w* P407/1.5% MC/0.5% *w*/*w* PEC) mixture obtained at time 0 and after 3 week storage at 4 °C. (a) viscosity profile measured at 25 °C, (b) viscosity profiles measured at 37 °C (mean values ± s.d.; *n* = 3). Figure S2: Viscosity curves of 4cM (15% *w*/*w* P407/0.75% MC/0.5% *w*/*w* PEC/0.25% *w*/*w* XYL) mixture obtained at time 0 and after 3 week storage at 4 °C. (a) viscosity profile measured at 25 °C, (b) viscosity profiles measured at 37 °C (mean values ± s.d.; *n* = 3).
