# Supplementary material for: Development of a Mucoadhesive In Situ Gelling Formulation for the Delivery of Lactobacillus gasseri into Vaginal Cavity
Source: Pharmaceutics. 2019 Oct 3;11(10):511. doi: 10.3390/pharmaceutics11100511 (PMC6836057; doi:10.3390/pharmaceutics11100511)
Supplement: Supplementary file 1 [file pharmaceutics-11-00511-s001.pdf]

Supplementary Materials: Development of a Mucoadhesive *in Situ* Gelling Formulation for the Delivery of *Lactobacillus gasseri* into Vaginal Cavity

Barbara Vigani, Angela Faccendini, Silvia Rossi, Giuseppina Sandri, Maria Cristina Bonferoni, Pietro Grisoli and Franca Ferrari

Preliminary Physical Stability Test

Both 3cM (15% w/w P407/1.5% MC/0.5% w/w PEC) and 4cM (15% w/w P407/0.75% w/w MC/0.5% w/w PEC/0.25% w/w XYL) mixtures were stored at 4 °C for 3 weeks. After 3 weeks storage, they were subjected to viscosity measurements at 25°C and 37°C. The viscosity measurements were carried out by means of a rotational rheometer (MCR 102, Anton Paar, Turin, Italy) equipped with a cone plate combination (CP50-1, diameter=50 mm; angle=1°) as measuring system.

Sample viscosity was measured by applying increasing shear rate values in the range 10- 300 s^{-1}.

In Figure 1S viscosity curves of 3cM mixtures obtained at 25°C (a) and at 37°C (b) upon storage at 4°C are compared with that obtained at time 0 (t=0). The viscosity curve obtained at 25°C upon storage is superimposable on that obtained at time 0 (Figure 1S a), indicating that the gel is stable for the period of time considered. Moreover, the viscosity curves obtained at 37°C (at time 0 and upon storage) are comparable. It indicates that, upon storage, the formulation maintains its capability to gelify at 37°C, forming a gel having the same viscosity of that observed at 37°C for the formulation freshly prepared (time 0) (Figure 1S b).

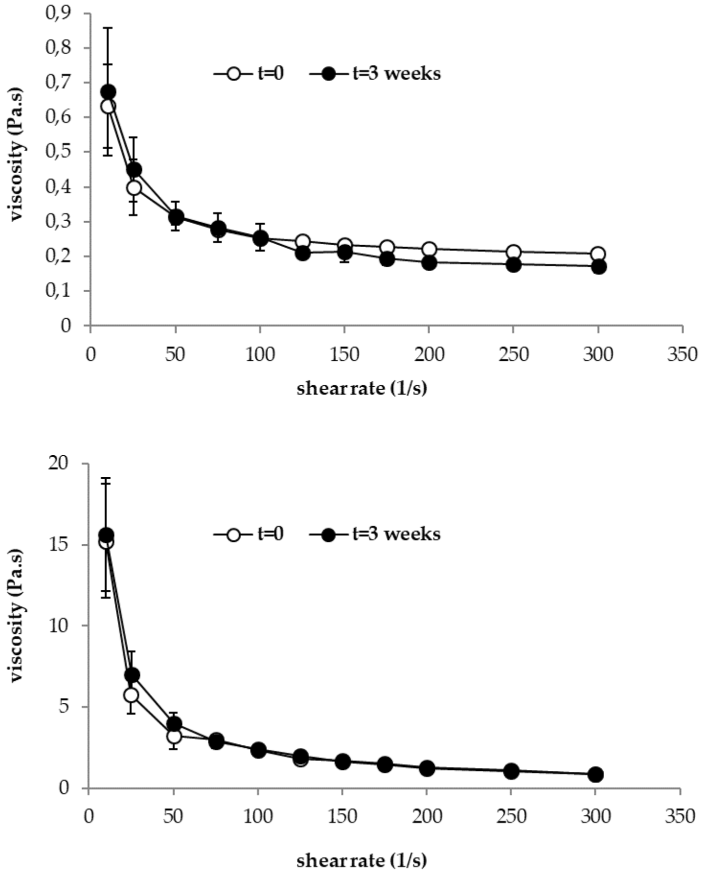

Figure S1. Viscosity curves of 3cM (15% w/w P407/1.5% MC/0.5% w/w PEC) mixture obtained at time 0 and after 3 week storage at 4°C a) viscosity profile measured at 25°C, b) viscosity profiles measured at 37°C (mean values ± s.d.; n=3).

4cM formulation shows the same behaviour of 3cM one (Figure 2S).

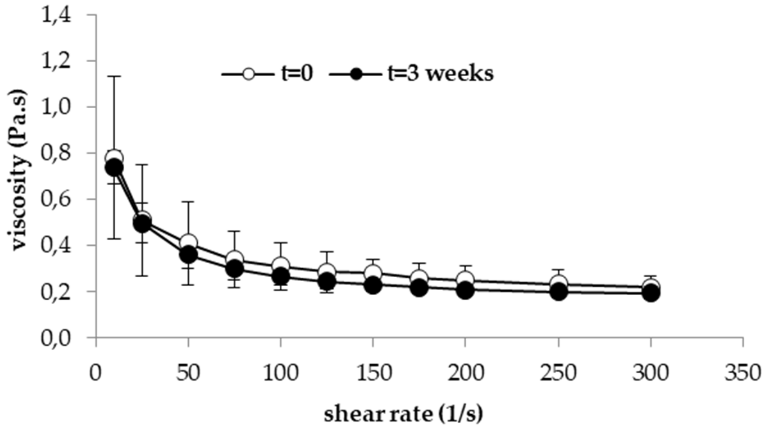

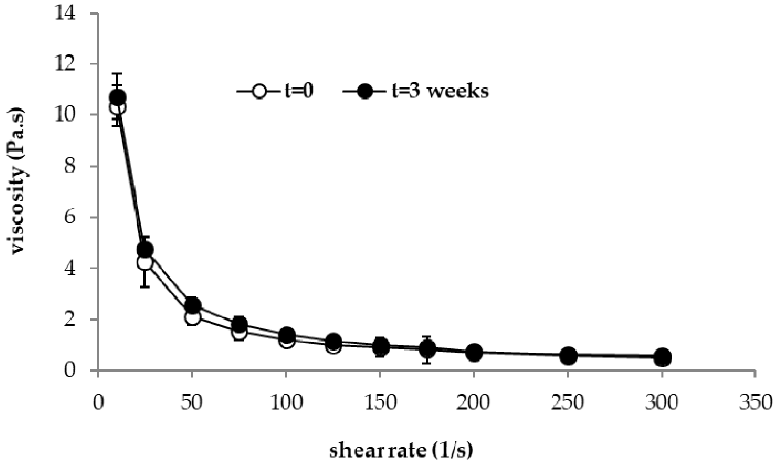

Figure S2. Viscosity curves of 4cM (15% w/w P407/0.75% MC/0.5% w/w PEC/0.25% w/w XYL) mixture obtained at time 0 and after 3 week storage at 4°C a) viscosity profile measured at 25°C, b) viscosity profiles measured at 37°C (mean values ± s.d.; n=3).
